# Clinical Outcomes of Coronary Artery Perforation Treated With Covered Stents: The Impact of Intravascular Ultrasound Guidance in a Contemporary Cohort

**DOI:** 10.1016/j.jscai.2025.103919

**Published:** 2025-10-14

**Authors:** Omar Sami Abdelhai, Ahmad Jabri, Sant Kumar, Ahmed Ghoneem, David Gelovani, Adnan Halboni, Mohammad Memon, John Major, Parsa Kamalipour, Ismail Jiazuldin, Brittany Fuller, Brian O’Neill, Herbert D. Aronow, Mir Babar Basir, William W. O’Neill, Mohammad Alqarqaz

**Affiliations:** a Division of Cardiovascular Medicine, Henry Ford Hospital, Detroit, Michigan; b Department of Cardiovascular Medicine, University of Pittsburgh Medical Center, Harrisburg, Pennsylvania

**Keywords:** coronary artery perforation, covered stents, intravascular ultrasound, percutaneous coronary intervention, target vessel revascularization

## Abstract

**Background:**

Coronary artery perforation (CAP) during percutaneous coronary intervention carries significant morbidity and mortality; however, data are limited regarding the specific role of intravascular ultrasound (IVUS) in evaluating and guiding management after CAP occurs and whether this approach improves clinical outcomes.

**Methods:**

This study consisted of a retrospective analysis of all patients who underwent percutaneous coronary intervention and experienced CAP treated with covered stents at a single tertiary care hospital between December 2014 and January 2024. The primary outcome was target vessel revascularization (TVR). Secondary outcomes included mortality, myocardial infarction, in-stent restenosis, and emergency cardiac surgery. Multivariable logistic regression was used to assess the association between IVUS and outcomes.

**Results:**

Between 2014 and January 2024, 127 cases of CAP treated using covered stents were identified. Of these, 34 patients (26.8%) underwent IVUS-guided covered stent optimization, whereas 93 patients (73.2%) did not undergo IVUS. Patients in the IVUS group were older (75.1 ± 8.4 vs 61.0 ± 10.3 years; *P* < .001) and predominantly women (55.9% vs 31.2%; *P* = .020). The IVUS was more frequently used for left anterior descending artery perforations (64.7% vs 36.6%; *P* = .009). IVUS was associated with a reduced risk of TVR during an average follow-up of 60.3 months (adjusted odds ratio, 0.04; 95% CI, 0.02-0.56; *P* = .031). Other outcomes, including mortality and repeat myocardial infarction, were similar between the 2 groups.

**Conclusions:**

The use of IVUS in patients requiring covered stents for CAP treatment, despite higher procedural complexity and a higher prevalence of severe perforations, was associated with significantly reduced TVR. This suggests a potential role for IVUS in improving clinical outcomes following CAP.

## Introduction

Coronary artery perforation (CAP) is a rare but severe complication encountered during percutaneous coronary intervention (PCI), with reported incidence rates ranging from 0.3% to 0.4% of all PCI procedures.^1^ Acutely, CAP can lead to life-threatening complications such as cardiac tamponade, myocardial infarction (MI), and emergency surgical interventions, contributing to in-hospital mortality rates of 5% to 15%.^1^^,^^2^ Long-term implications of CAP are also considerable, with increased risks of recurrent MI, repeat PCI procedures, target vessel revascularization (TVR), and reduced overall survival.^3^ The morbidity associated with CAP, particularly in cases requiring covered stent implantation or other emergency interventions, underscores the importance of timely recognition and optimal management strategies to mitigate subsequent cardiovascular events.

Several previous studies have attempted to characterize outcomes and predictors associated with CAP. These studies have identified advanced age, female sex, chronic kidney disease (CKD), hypertension, previous coronary artery bypass graft (CABG), and procedural complexity—such as PCI involving calcified lesions, left main or proximal left anterior descending artery (LAD) lesions, or chronic total occlusions (CTOs)—as significant predictors of CAP occurrence and poor outcomes.^1^^,^^4^^,^^5^ However, there is a paucity of contemporary data evaluating the impact of adjunctive intravascular imaging modalities.^6^, ^7^, ^8^ Although evidence indicates that IVUS-guided PCI can reduce PCI-related CAP^8^ or assist in its detection,^7^ data specifically on IVUS impact on patient outcomes after CAP treated with covered stents is limited. Given these gaps in current knowledge, our study aimed to assess the potential clinical advantages of using IVUS to optimize management of CAP during PCI.

## Materials and methods

### Study population

This study consisted of a retrospective analysis of all patients who underwent PCI and developed CAP that required treatment with at least 1 covered stent at a single tertiary care hospital between December 2014 and January 2024. Data were manually extracted from the hospital’s electronic health record. The extracted data included patient demographic characteristics, comorbidities, clinical presentation, angiographic and procedural data, complications, and in-hospital adverse events. Our institutional review board approved the study. Given its retrospective nature, the requirement for informed consent was waived.

### Outcomes and definitions

The primary outcome was TVR. Secondary outcomes analyzed included in-hospital and late mortality, repeat MI, in-stent restenosis (ISR), and the need for emergency procedures such as pericardiocentesis or cardiac surgery.

The definitions of the procedural variables were defined using the National Cardiovascular Data Registry CathPCI Registry (version 5.0 dictionary). Death, MI, and stroke were determined following the guidelines of the Academic Research Consortium 2 consensus document.

Patients experiencing CAP were divided into 2 groups based on whether IVUS was used to optimize covered stent placement for perforation management. The use of IVUS was at the operator’s discretion.

### Statistical analysis

Continuous variables were presented as mean ± SD and compared using the independent sample *t* test, Mann-Whitney *U* test, or analysis of variance, as appropriate. Categorical variables were presented as absolute numbers and percentages and compared using χ^2^ or Fisher exact test, as appropriate.

Univariate logistic regression analysis was performed to test the association between baseline covariates and TVR, with results expressed as odds ratio (OR) with 95% CI. Multivariable logistic regression for TVR was performed to adjust for potential confounders using variables with a *P* value of <.10 on univariable analysis and clinical plausibility. The final model used for adjustment included the following variables: age, sex, body mass index, diabetes mellitus, hypertension, hyperlipidemia, CKD, smoking history, previous MI, previous CABG, left ventricular ejection fraction of <50%, mechanical circulatory support (MCS) use, LAD coronary perforation, left main artery coronary perforation, left circumflex artery coronary perforation, right coronary artery coronary perforation, CTO, bifurcation, ISR, complex lesion C, protamine administration, use of atherectomy or intravascular lithotripsy, and indication of PCI (acute coronary syndrome vs nonacute coronary syndrome).

## Results

### Baseline characteristics

Between December 2014 and January 2024, we identified 127 cases of CAP during PCI that required treatment with at least 1 covered stent. Of these, IVUS was used to assess vessel size and optimize covered stent placement in 34 patients (26.8%), whereas IVUS was not used in 93 patients (73.2%). As shown in Table 1, patients in the IVUS group were significantly older (75.1 ± 8.4 years) than those in the no IVUS group (61.0 ± 10.3 years; *P* < .001). Eighty-nine patients (70%) received a Papyrus covered stent (Biotronik) and 38 patients (30%) a GRAFTMASTER covered stent (Abbott) (Supplemental Table S1). IVUS use was more frequent in women (55.9% vs 31.2%; *P* = .020). Other baseline characteristics such as body mass index, diabetes mellitus, hypertension, hyperlipidemia, CKD, smoking history, previous MI, previous CABG, and left ventricular ejection fraction did not significantly differ between the 2 groups.

**Table 1:** Baseline characteristics.

Characteristic	IVUS (n = 34)	No IVUS (n = 93)	*P*
Age, y	75.1 ± 8.4	61.0 ± 10.3	<.001
Female sex	19 (55.9)	29 (31.2)	.02
Body mass index, kg/m^2^	28.1 ± 8.5	29.4 ± 7.2	.43
Diabetes mellitus	20 (58.8)	48 (51.6)	.60
Hypertension	32 (94.1)	85 (91.4)	.90
Hyperlipidemia	28 (82.4)	80 (86.0)	.82
Chronic kidney disease	20 (58.8)	43 (46.2)	.29
Smoker	21 (61.8)	61 (65.6)	.85
Previous myocardial infarction	14 (41.2)	48 (51.6)	.40
Previous CABG	7 (20.6)	35 (37.6)	.11
LVEF, %	49.9 ± 17.0	48.0 ± 14.7	.57
LVEF <50%	9 (26.5)	40 (43.0)	.14
Presentation			
Stable angina	21 (61.8)	60 (64.5)	.94
NSTE-ACS	12 (35.3)	30 (32.3)	.91
STEMI	1 (2.9)	3 (3.2)	>.99

Values are n (%) or mean ± SD.

CABG, coronary artery bypass graft; IVUS, intravascular ultrasound; LVEF, left ventricular ejection fraction; NSTE-ACS, non–ST-elevation acute coronary syndrome; STEMI, ST-segment elevation myocardial infarction.

### Angiographic and procedural characteristics

**Figure 1:**
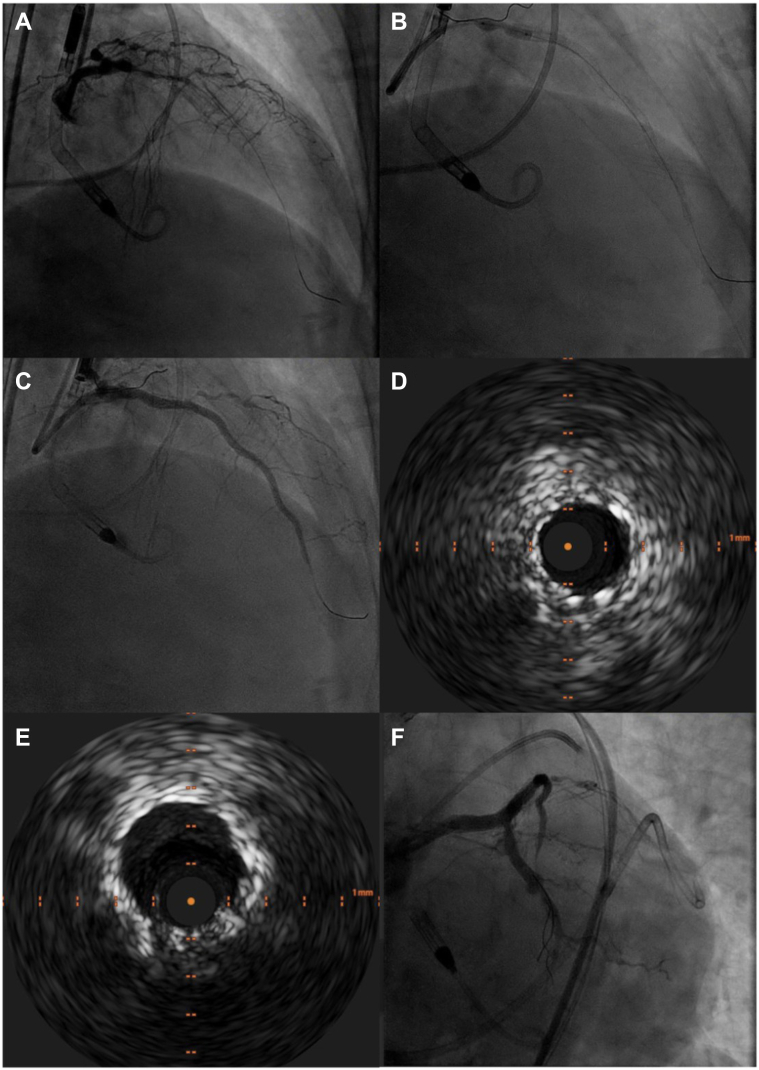
**Management of coronary artery perforation in proximal left anterior descending (LAD) artery.** (**A**) Baseline angiogram showing lesion in proximal LAD. (**B**) Balloon inflation resulting in coronary artery perforation. (**C**) Covered stent placement to manage the perforation. (**D**) Intravascular ultrasound (IVUS) visualization of the covered stent before dilation. (**E**) IVUS-guided postdilation of the covered stent. (**F**) Final angiogram demonstrating resolution of perforation after IVUS-guided postdilation of the covered stent.

As depicted in Table 2, IVUS was more commonly used in CAP involving the LAD artery (64.7% vs 36.6%; *P* = .009), while right CAPs were more common in the non-IVUS group (44.1% vs 20.6%; *P* = .027). Figure 1 illustrates an example of a CAP occurring during balloon angioplasty of a proximal LAD lesion, with IVUS subsequently used to evaluate the expansion of the implanted covered stent. There was no significant difference between groups in CTO-related perforations (*P* = .192), calcification severity (*P* = .625), ISR (*P* = .920), bifurcation lesions (*P* = .980), or MCS use (*P* = .201). Characteristics of covered stents and balloon sizes are detailed in Supplemental Table S2.

**Table 2:** Angiographic and procedural characteristics.

Characteristic	IVUS (n = 34)	No IVUS (n = 93)	*P*
Vessel			
Left anterior descending artery	22 (64.7)	34 (36.6)	.009
Left circumflex artery	4 (11.8)	12 (12.9)	>.99
Ramus	0 (0)	2 (2.2)	.96
Right coronary artery	7 (20.6)	41 (44.1)	.03
Left main artery	1 (2.9)	4 (4.3)	>.99
Chronic total occlusion	15 (44.1)	55 (59.1)	.19
Ostial	16 (47.1)	43 (46.2)	>.99
Moderate or severe calcification	32 (94.1)	83 (89.2)	.63
In-stent restenosis	1 (2.9)	5 (5.4)	.92
Bifurcation	11 (32.4)	28 (30.1)	.98
Mechanical circulatory support use	9 (26.5)	38 (40.9)	.20
Cardiac tamponade requiring pericardiocentesis	18 (52.9)	26 (28.0)	.012
Complex lesion	34 (100)	88 (94.6)	.39
No. of covered stents	1.3 ± 0.5	1.5 ± 0.9	.12
Covered stent diameter, mm	3.5 ± 0.7	3.9 ± 2.8	.21
Largest predilation balloon diameter, mm	2.4 ± 1.7	2.7 ± 1.7	.38
Largest postdilation balloon diameter, mm	3.8 ± 0.9	3.8 ± 0.8	>.99

Values are n (%) or mean ± SD.

IVUS, intravascular ultrasound.

No significant differences were observed in the causes of perforation between the 2 groups. Ellis type 3 perforations were the most common type in both groups (73.5% [IVUS] vs 80.6% [no IVUS]; *P* = .533) (Table 3).

**Table 3:** Coronary perforation characteristics.

Characteristic	IVUS (n = 34)	No IVUS (n = 93)	*P*
Cause of perforation			
Initial percutaneous transluminal coronary angioplasty	9 (26.5)	18 (19.4)	.53
Stent^a^	8 (23.5)	34 (36.6)	.24
Atherectomy	4 (11.8)	3 (3.2)	.15
Lithotripsy	0 (0)	2 (2.2)	.96
Postdilation	10 (29.4)	28 (30.1)	>.99
Wire	3 (8.8)	7 (7.5)	>.99
Other	0 (0)	1 (1.1)	>.99
Ellis type			
Type 1	3 (8.8)	1 (1.1)	.10
Type 2	6 (17.6)	17 (18.3)	>.99
Type 3	25 (73.5)	75 (80.6)	.53

IVUS, intravascular ultrasound.

a Stent indicates coronary perforation identified angiographically immediately following stent deployment, typically due to vessel overstretching or oversizing.

### Clinical outcomes

During a mean follow-up period of 60.3 months, IVUS use was associated with significantly lower rates of TVR (5.9% [IVUS] vs 24.7% [no IVUS]; *P* = .039). Patients in the IVUS group had higher rates of cardiac tamponade requiring pericardiocentesis (52.9% vs 28.0%; *P* = .012). Other outcomes, such as in-hospital mortality, late MI, covered stent thrombosis, repeat angiography, emergency cardiac surgery, MCS, and protamine use were similar between groups. There was a trend toward reduced need for a second covered stent in the IVUS group, but it did not reach statistical significance (Table 4). Clinical outcomes based on the type of covered stent used are shown in Supplemental Table S3.

**Table 4:** Clinical outcomes.

Outcome	IVUS (n = 34)	No IVUS (n = 93)	*P*
In-hospital mortality	6 (17.6)	23 (24.7)	.58
Posthospitalization mortality	2 (5.9)	11 (11.8)	.54
Covered stent thrombosis	2 (5.9)	8 (8.6)	.92
In-stent restenosis	2 (5.9)	9 (9.7)	.78
Repeat myocardial infarction	3 (8.8)	7 (7.5)	.98
Repeat angiography	16 (47.1)	51 (54.8)	.64
Repeat percutaneous coronary intervention	9 (26.5)	29 (31.2)	.82
Target vessel revascularization	2 (5.9)	23 (24.7)	.04
Emergency cardiac surgery	1 (2.9)	0 (0)	.59
Mechanical circulatory support	9 (26.5)	38 (40.9)	.23
Protamine use	9 (26.5)	32 (34.4)	.58
Need for second covered stent	9 (26.5)	35 (37.6)	.38

IVUS, intravascular ultrasound.

### Predictors of target vessel revascularization

On unadjusted analysis, IVUS use significantly reduced the risk of TVR (OR, 0.03; 95% CI, 0.01-0.26; *P* = .002), a finding that remained significant in adjusted analysis (adjusted OR, 0.04; 95% CI, 0.02-0.56; *P* = .031). Diabetes mellitus (OR, 4.54; 95% CI, 1.38-11.0; *P* = .013) and MCS use (OR, 2.15; 95% CI, 1.18-6.02; *P* = .022) were significantly associated with higher TVR rates on unadjusted analysis; diabetes remained significant also in adjusted analysis (adjusted OR, 2.05; 95% CI, 1.89-4.15; *P* = .022) (Table 5).

**Table 5:** Predictors of target vessel revascularization after percutaneous coronary intervention complicated by coronary perforation.

Variable	Univariate			Multivariable		
	OR	95% CI	*P*	OR	95% CI	*P*
No IVUS	Reference			Reference		
IVUS	0.03	0.01-0.26	.002	0.04	0.02-0.56	.03
Age	1.15	0.89-1.21	.37			
Female sex	0.51	0.15-1.71	.28			
Body mass index	1.21	0.71-1.67	.20			
Diabetes mellitus	4.54	1.38-11.0	.013	2.05	1.89-4.15	.02
Hypertension	1.91	0.22-10.61	.56			
Hyperlipidemia	1.10	0.18-2.19	.65			
Chronic kidney disease	1.61	0.48-2.89	.37			
Smoking history	1.19	0.31-2.55	.38			
Previous MI	1.81	0.29-2.16	.64			
Previous CABG	1.24	0.44-3.46	.68			
LVEF <50%	1.16	0.42-3.26	.77			
MCS use	2.15	1.18-6.02	.02	1.39	0.76-2.04	.17
LAD coronary perforation	2.76	1.96-7.83	.04	1.32	0.67-4.60	.14
Left main coronary perforation	1.50	0.48-5.10	.91			
Left circumflex artery perforation	1.31	1.03-2.59	.28			
Right coronary artery perforation	0.74	0.25-2.18	.59			
Chronic total occlusion	3.27	1.98-8.91	.03	1.31	0.53-6.59	.75
Bifurcation	1.38	1.01-1.44	.16			
In-stent restenosis	1.28	0.49-7.10	.94			
Complex lesion C	1.14	1.01-4.92	.04	1.01	0.37-2.10	.55
Protamine administration	0.89	0.30-2.61	.68			
Atherectomy/IVL use	1.97	0.17-2.98	.59			
Indication for PCI						
ACS	3.52	1.94-8.15	.018	1.04	0.14-3.78	.65
Non-ACS	0.66	0.17-2.58	.56			

ACS, acute coronary syndrome; CABG, coronary artery bypass graft; IVL, intravascular lithotripsy; IVUS, intravascular ultrasound; LAD, left anterior descending artery; LVEF, left ventricular ejection fraction; MCS, mechanical circulatory support; MI, myocardial infarction; OR, odds ratio; PCI, percutaneous coronary intervention.

## Discussion

The key findings of our study are as follows: (1) use of IVUS to optimize covered stent placement for management of CAP was associated with significantly lower rates of TVR, and (2) despite higher incidence of cardiac tamponade requiring pericardiocentesis in the IVUS group, outcomes such as in-hospital mortality, repeat MI, covered stent thrombosis, repeat angiography, emergency cardiac surgery, MCS, and protamine use were similar between groups (Central Illustration).

**Central Illustration:**
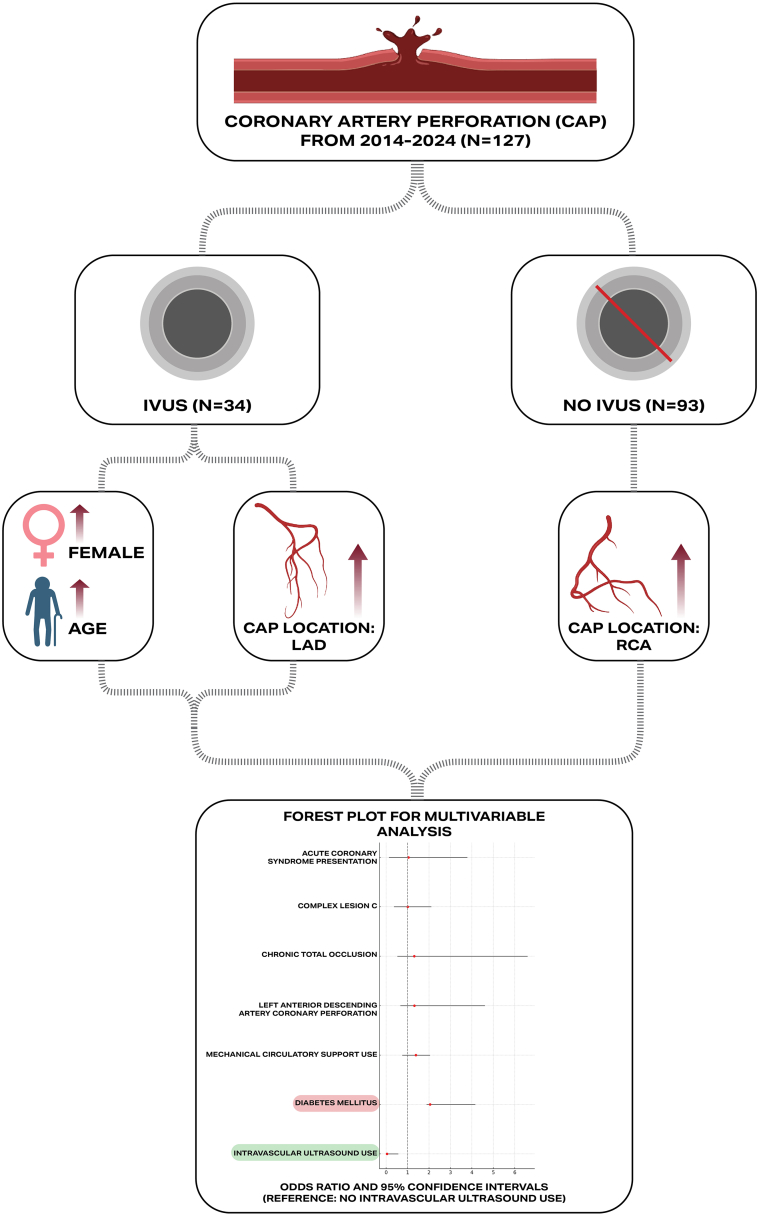
**Representative case of proximal LAD (Left anterior descending ) cover stent IVUS guided optimization.** Flowchart illustrating the distribution of coronary artery perforation (CAP) cases from 2014 to 2024, highlighting differences based on intravascular ultrasound (IVUS) usage, patient demographic characteristics, and perforation location, along with a forest plot summarizing the multivariable analysis of predictors for CAP occurrence.

Sawayama et al^8^ assessed whether IVUS-guided PCI reduced the risk of PCI-related CAP in 22,368 procedures, which resulted in 368 CAPs. Although IVUS guidance was associated with a significantly reduced overall perforation risk, there are limited data on the impact of IVUS use on clinical outcomes in patients with coronary perforation who require covered stent placement.^8^ Nairooz et al^9^ used the National Cardiovascular Data Registry CathPCI Registry to examine in-hospital outcomes for patients with CAP and those without CAP. The most significant predictors of adverse events among patients with CAP were cardiogenic shock, cardiac arrest, and the use of atherectomy. Nevertheless, detailed procedural data, including the cause of CAP and the absence of a core laboratory to validate CAP cases, were lacking.^9^ Our study addresses previous limitations by specifically evaluating the impact of IVUS-guided covered stent optimization—suggesting an advantage in reducing TVR—and provides detailed procedural insights into the outcomes of CAP. For example, the observation that patients in the IVUS group were older and more frequently female likely reflects a higher underlying procedural complexity and perceived risk, as advanced age and female gender have been previously identified as risk factors for CAP.^9^^,^^10^ This suggests operators may have selectively used IVUS guidance in more challenging cases, potentially aiming to mitigate the increased procedural risks associated with these patient characteristics. However, we acknowledge that the observed lower TVR rate in the IVUS group should be interpreted cautiously, given our study’s limited sample size and statistical power. The significance of this finding may be sensitive to minor changes in event rates, and thus, it may reflect chance rather than a robust clinical difference.

IVUS’s favorable impact on TVR rates may be related to several factors. First, using IVUS at baseline to accurately determine vessel size may guide the selection of the appropriate covered stent size. Perforations often result in hypotension and the use of vasopressor therapy, which can lead to coronary spasm. IVUS may help ensure proper covered stent expansion and apposition, especially when deployed at low inflation pressures, given concerns from operators over the potential for worsening perforation and extravasation. Moreover, IVUS assessment can identify other mechanical complications, such as intramural hematomas and stent edge dissections, adjacent to the covered stent, guiding further management strategies.^11^ Zebrauskaite et al^6^ emphasized the utility of IVUS in sizing and optimizing covered stents, which can reduce the risk of stent thrombosis and restenosis, factors known to drive the need for subsequent revascularization procedures.

Current guidelines from the American College of Cardiology, American Heart Association, and Society for Cardiovascular Angiography & Interventions recommend the routine use of intravascular imaging, including IVUS, to optimize procedural outcomes and minimize major adverse cardiac events during complex PCI; however, there are no specific recommendations regarding IVUS utilization for managing CAP.^12^ Considering the frequent necessity for deploying covered stents in severe CAP cases, there is a heightened risk of subsequent covered stent thrombosis, a complication that is increasing.^13^ Our study demonstrates the potential benefit of IVUS in optimizing covered stent placement following CAP. Although the majority of perforations in our group were severe (Ellis type 3), where prompt management is essential due to the potential for serious complications, IVUS guidance was still linked to positive clinical outcomes. This suggests that IVUS can enhance the placement of covered stents and procedural decision making in complex situations.

### Study limitations

Our study has several limitations inherent to its retrospective and observational design. First, the use of IVUS was left to the operator’s discretion, potentially introducing selection bias, as evidenced by greater procedural complexity and higher rates of LAD perforation and cardiac tamponade in the IVUS group. However, a manual chart review was conducted to confirm that IVUS imaging was specifically performed to guide covered stent placement following coronary perforation. Second, despite adjusting for multiple confounding variables in the multivariable analysis, residual confounding may still persist. Owing to the relatively small sample size, propensity score matching was not feasible. Third, the relatively small sample size and single center setting limit the generalizability of our findings. Our study included a small cohort from a single center, and despite demonstrating lower TVR with IVUS, other critical outcomes such as mortality, ISR, repeat MI, and emergency procedures did not differ, potentially reflecting the high overall morbidity associated with coronary perforations. Fourth, historical bias may have influenced our results, as IVUS utilization increased in recent years. This temporal trend could reflect evolving operator practices and greater familiarity with IVUS technology, potentially affecting the observed outcomes (Supplemental Figure S1). Finally, the absence of standardized IVUS protocols across operators and procedures might have influenced both the detection of vessel injury and subsequent management decisions.

## Conclusion

In this contemporary cohort of patients undergoing PCI complicated by CAP requiring treatment with covered stents, the use of IVUS for assessing and guiding treatment postperforation was associated with significantly lower rates of TVR. These results suggest a clinical benefit for IVUS guidance following CAP; however, these findings should also be interpreted cautiously given the study’s limited sample size. Further prospective research and guideline recommendations are warranted to clarify the routine role of IVUS in the management of CAP.
