# Supplementary material for: Clinical Outcomes of Coronary Artery Perforation Treated With Covered Stents: The Impact of Intravascular Ultrasound Guidance in a Contemporary Cohort
Source: J Soc Cardiovasc Angiogr Interv. 2025 Oct 14;4(11):103919. doi: 10.1016/j.jscai.2025.103919 (PMC12664623; doi:10.1016/j.jscai.2025.103919)
Supplement: Supplemental Tables and Figures [file mmc1.docx]

**Supplementary Materials**

| **Supplemental Table 1. Baseline characteristics by covered stent type** | | |
| --- | --- | --- |
|  | Papyrus  N = 89 | GraftMaster  N = 38 |
| Age | 72.0±9.7 | 74.1±8.9 |
| Female | 37 (41.6) | 12 (31.6) |
| Body mass index | 28.7±8.2 | 29.7±6.1 |
| Diabetes mellitus | 53 (60.0) | 15 (39.5) |
| Hypertension | 83 (93.3) | 35 (92.1) |
| Hyperlipidemia | 78 (87.6) | 31 (81.6) |
| Chronic kidney disease | 47 (52.8) | 17 (44.7) |
| Smoking history | 60 (67.4) | 22 (57.9) |
| Prior MI | 48 (53.9) | 14 (36.8) |
| Prior CABG | 32 (36.0) | 10 (26.3) |
| LVEF < 50% | 33 (37.1) | 16 (42.1) |
| LVEF (%) | 48.3±15.3 | 49.3±15.3 |
| Indication of PCI |  |  |
| *Stable CAD* | 55 (61.8) | 20 (52.6) |
| *NSTE-ACS* | 31 (34.8) | 17 (44.7) |
| *STEMI* | 3 (3.4) | 1 (2.6) |
| Abbreviations: CABG = coronary artery bypass graft; CAD = coronary artery disease; LVEF = left ventricular ejection fraction; MI = myocardial infarction; NSTE-ACS = Non ST-elevation acute coronary syndrome; PCI = percutaneous coronary intervention; STEMI = ST-elevation myocardial infarction | | |

| **Supplemental Table 2. Angiographic and procedural characteristics by covered stent type** | | |
| --- | --- | --- |
|  | Papyrus  N = 89 | GraftMaster  N = 38 |
| Coronary artery perforation location |  |  |
| *LAD* | 40 (44.9) | 16 (42.1) |
| *Left circumflex artery* | 13 (14.6) | 3 7.9) |
| *Ramus intermedius* | 2 (2.2) | 0 (0) |
| *Right coronary artery* | 33 (37.1) | 15 (39.5) |
| *Left main* | 1 (1.1) | 4 (10.5) |
| *Saphenous vein graft* | 0 (0) | 0 (0) |
| Chronic total occlusion | 52 (58.4) | 18 (47.4) |
| Ostial location | 38 (42.7) | 21 (55.3) |
| Moderate to severe calcification | 78 (87.6) | 37 (97.4) |
| Complex lesion C | 86 (96.6) | 36 (94.7) |
| Number of covered stents needed | 1.5±0.8 | 1.5±0.9 |
| Diameter of first covered stent | 3.9±2.9 | 3.6±0.7 |
| Length of first covered stent | 22.2±4.7 | 20.4±4.2 |
| Bifurcation lesion | 26 (29.2) | 13 (34.2 |
| Abbreviations: LAD = left anterior descending artery | | |

| **Supplemental Table 3. Outcomes by Covered Stent Type** | | |
| --- | --- | --- |
|  | Papyrus  N = 89 | GraftMaster  N = 38 |
| In-hospital mortality | 17 (19.1) | 12 (31.6) |
| Post-hospitalization mortality | 10 (11.2) | 3 (7.9) |
| Covered stent thrombosis | 7 (7.9) | 2 (5.3) |
| In-stent restenosis | 8 (9.0) | 5 (13.2) |
| Repeat myocardial infarction | 6 (6.7) | 4 (10.5) |
| Repeat angiography | 48 (53.9) | 18 (47.4) |
| Repeat PCI | 29 (32.6) | 11 (28.9) |
| TLR | 12 (13.5) | 4 (10.5) |
| TVR | 18 (20.2) | 4 (10.5) |
| Emergency cardiac surgery | 1 (1.1) | 0 (0) |
| Cardiac tamponade requiring pericardiocentesis | 30 (33.7) | 14 (36.8) |
| MCS | 26 (29.2) | 21 (55.3) |
| Protamine use | 26 (29.2) | 15 (39.5) |
| Abbreviations: MCS = mechanical circulatory support; PCI = percutaneous coronary intervention; TLR = target lesion revascularization; TVR = target vessel revascularization | | |
